# Supplementary material for: How self-efficacy beliefs in dealing with negative emotions are associated to negative affect and to life satisfaction across gender and age
Source: PLoS One. 2020 Nov 18;15(11):e0242326. doi: 10.1371/journal.pone.0242326 (PMC7673490; doi:10.1371/journal.pone.0242326)
Supplement: S1 Models — (PPTX) [file pone.0242326.s004.pptx]

## Slide 1
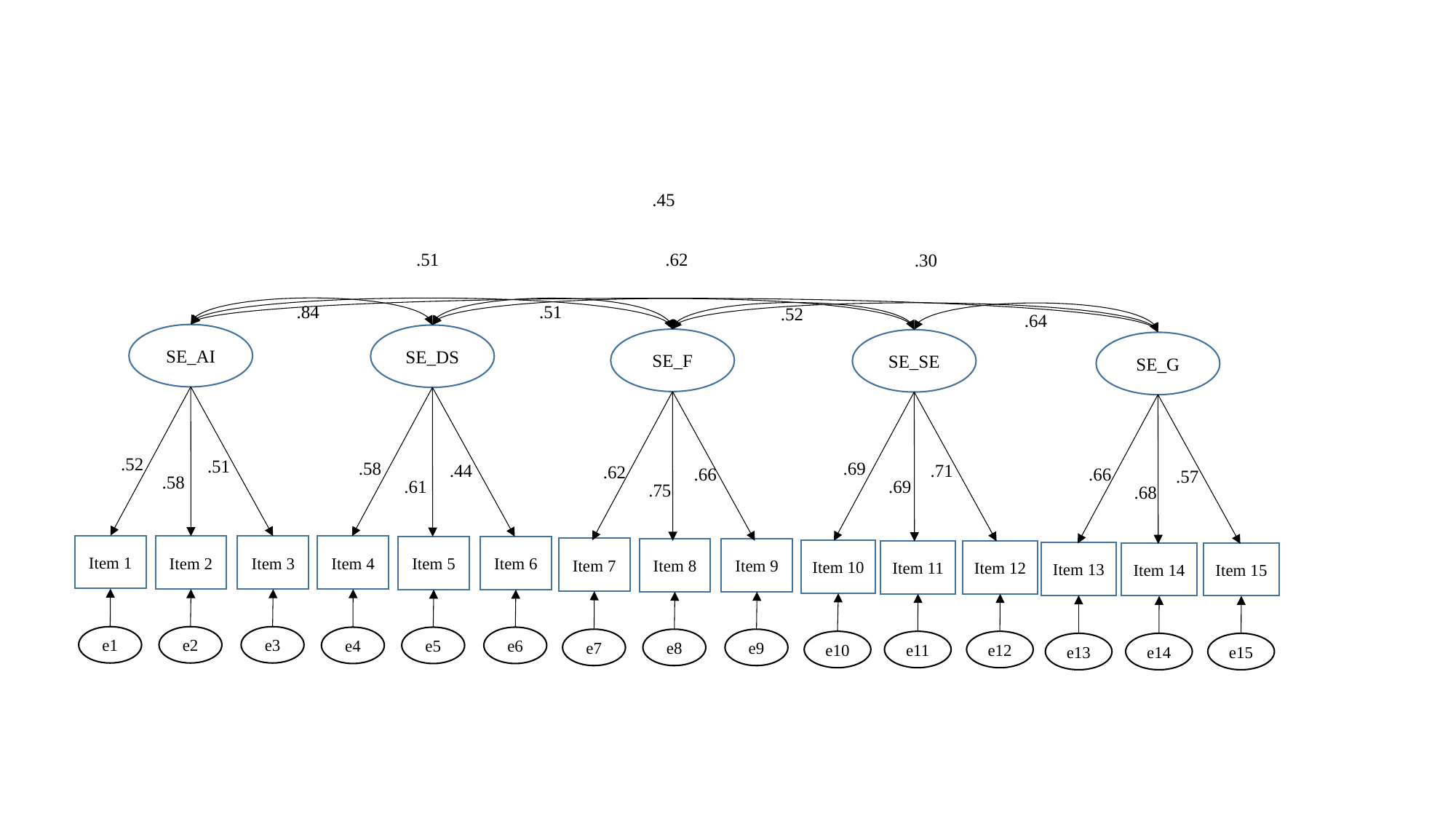

.45
.51
.62
.30
.84
.51
.52
.64
SE_AI
SE_DS
SE_F
SE_SE
SE_G
.52
.51
.58
.69
.44
.71
.62
.66
.66
.57
.58
.61
.69
.75
.68
Item 1
Item 2
Item 3
Item 4
Item 5
Item 6
Item 7
Item 8
Item 9
Item 10
Item 11
Item 12
Item 13
Item 14
Item 15
e1
e2
e3
e4
e5
e6
e7
e8
e9
e10
e11
e12
e13
e14
e15
