# Supplementary material for: How self-efficacy beliefs in dealing with negative emotions are associated to negative affect and to life satisfaction across gender and age
Source: PLoS One. 2020 Nov 18;15(11):e0242326. doi: 10.1371/journal.pone.0242326 (PMC7673490; doi:10.1371/journal.pone.0242326)
Supplement: Attachment — Submitted filename: Review PONE-D-20-00499.docx [file pone.0242326.s005.docx]

The goal of the present study was to how how people’s efficacy beliefs about managing negative emotions impact experienced negative affect and well-being in an adult Spanish sample. In particular, the authors aim to establish measurement invariance in the Spanish sample across age and gender, and examine construct validity of the measure in a Spanish sample by relating the MNESRES to outcomes of adaptation. The study is well-conducted, the analyses rigorous, and the conclusions sound. There are only some minor changes I would recommend, as detailed below:

INTRODUCTION:

The first paragraph discusses social cognitive theory of personality, which feels a bit off without first introducing emotional regulatory self-efficacy. A re-ordering of this paragraph would help the reader make the connection between social cognitive theory of personality and the primary focus of the paper—emotional regulatory self-efficacy—more readily. The introduction overall seems to focus very specifically on emotion regulation self-efficacy scale with one group of researchers. A broader discussion of how this measure fits into the literature on emotion regulation and self-efficacy would be helpful. For example, Ford & Gross 2019 Current Directions paper discusses this in some detail (see references).

METHODS:

Line 174: Please add example items for the MNESRES. Why is chronbach’s alpha not reported? Please add this info in.

Line 224, 229, and 233: Regressions instead of regression.

Line 264, 286-287, 296-297: Please provide the exact item wording.

DISCUSSION:

Discussion is well-written and thorough. However, more discussion of why negative self-conscious emotions didn’t relate to outcomes would strengthen the discussion. Furthermore, there is a lack of discussion about how positive emotions efficacy beliefs might explain the lack of findings for negative self-conscious emotions. For example, there is some evidence that positive self-conscious emotions, such as pride, may act as a mediator between self-efficacy beliefs and depression (Van Doren et al., 2019). The lack of inclusion of measures of positive self-conscious emotions may have impacted the findings.

References:

Ford, B. Q., & Gross, J. J. (2018). Emotion regulation: Why beliefs matter. Canadian Psychology, 59, 1–14. https://doi.org/10. 1037/cap0000142

Ford, B. Q., & Gross, J. J. (2019). Why beliefs about emotion matter: An emotion-regulation perspective. Current Directions in Psychological Science, 28, 74–81. https://doi.org/10.1177/0963721418806697

Van Doren, N., Tharp, J. A., Johnson, S. L., Staudenmaier, P. J., Anderson, C., & Freeman, M. A. (2019). Perseverance of effort is related to lower depressive symptoms via authentic pride and perceived power. Personality and Individual Differences, 137, 45–49.
