# Supplementary material for: How self-efficacy beliefs in dealing with negative emotions are associated to negative affect and to life satisfaction across gender and age
Source: PLoS One. 2020 Nov 18;15(11):e0242326. doi: 10.1371/journal.pone.0242326 (PMC7673490; doi:10.1371/journal.pone.0242326)
Supplement: Attachment — Submitted filename: Response to reviewers.docx [file pone.0242326.s006.docx]

***Reply to the issues raised by reviewer*** #1:

The manuscript deals with the question of the factorial structure and construct validity of the Multidimensional Negative Emotions Self-Regulatory Efficacy Scale (MNESRES), which measures self-efficacy beliefs in regulating discrete negative emotions. Overall, the manuscript seems scientifically rigorous to me. However, I have some questions and comments.

(1) Clear presentation of the aims, methods, & results

(a) Throughout the manuscript, the writing was not always very clear / concise.

**Our response:**

**As suggested from the reviewer we have revised the whole paper, in particular the text now presented in blue.**

(b) The introduction was too broad for me. The MNESRES, which should be front and center, is only mentioned at the end of the second page. I would have expected an introduction that focusses more on why this particular study is of importance. I would also have liked to have more information on why self-regulatory efficacy may be comparable across age groups and gender.

**Our response:**

**Thank you very much for your appreciations and interest, we have redrafted the introduction in order to make it clearer and more concise (see pages 2-6). By addressing the potential moderating effect of sex and age, our intention was to extend the generalizability of our results with regards the internal and external validity of the instrument.**

Also, the rationale for choosing the two outcomes (negative affect & satisfaction with life) was not clear to me.

**Our response:**

**This study aims to further validate the use of the MNESRES by addressing the degree to which individuals’ beliefs about their ability to control negative emotions is associated with important indicators of their well-being, such as Negative Affect (NA) and Life Satisfaction (SWL). Negative affect is thought to be strictly associated with the individuals’ capacity to manage their negative emotions, whereas life satisfaction has been regarded as a general indicator of the individuals’ well-being, which may be affected by their capacity to manage negative emotions to a varied extent.**

(c) Given that the authors stress the importance of shame and guilt, it seemed somewhat surprising to me that they expected stronger associations with negative affect (NA) and satisfaction with life (SWL) for the basic emotions.

**Our response:**

**Our reasoning is that the correct management of self-conscious/moral emotions is largely conditioned by the proper management of basic emotion. This is in accordance with previous findings suggesting a stronger impact of basic emotions than self-conscious/moral emotions (Caprara, 2013).**

**(2) Methods**

(a) Sample – The sample consisted of relatives and friends of undergraduate Psychology students. The authors may want to discuss that this is not ideal, given that participants were not independent from one another (e.g., couples, relatives, friends, etc.). Also, middle-aged men were oversampled and the distribution of age in the older age group seems skewed.

**Our response:**

**We have now addressed these limitations in the discussion (See page 24), where we explicitly acknowledge that we have used groups of convenience. Nevertheless, we believe that the robustness of our main findings are supported by their consistency across age and gender, in accordance with earlier results in another context.**

(b) MNESRES – I would help my understanding of the concept of self-regulatory efficacy to have a table with the MNESRES items or at least example items for each dimension.

**Our response:**

**We have now provided an example of an item for each of the MNESRES dimensions (see pages 8 and 9).**

(c) Negative affect – I was wondering why only NA was reported, but not PA. Given that the authors also assessed SWL, it would have made sense to report on subjective well-being (with the facets NA and PA for affective well-being, and SWL as the cognitive facet; see Diener). Alternatively, it would have been interesting to have measures of discrete emotions matched to the dimensions of the MNESRES as outcomes.

**Our response:**

**Since we focused on the individual’s perceived capacity to manage negative emotions, we examined their associations with the indicators of well-being’ that were most congruent with them. We do not rule out an association between self-efficacy beliefs regarding negative emotions and positive affect, yet we feel that this be best dealt with in a separate study.**

**Certainly it would be of interest to take advantage of the present findings and previous data showing how self-efficacy beliefs about the management of positive emotions are associated with positive affect (Gerbino et al., 2016) in order to further examine how self-efficacy beliefs about positive and negative emotions operate in concert.** Gerbino, M., Milion M., Alessandri, G., Eisenberg, N., Caprara, M., Kupfer, A., Pastorelli, C., & Vittorio Caprara, G. (2016, October 7). Self-Efficacy in Retrieving Positive Emotional Experience and Using Humor: A Validation Study of a New Instrument in Three Countries. *European Journal of Psychological Assessment*. Advance online publication. <http://dx.doi.org/10.1027/1015-5759/>a000356

(3) Analytic approach

(a) It was unclear to me why the three proposed models were tested. How were these models pre-selected?

**Our response:**

**This section has been entirely revised and rewritten as follows (see pages 9and 10):**

In order to investigate the factorial structure of the MNESRES, in accordance with previous findings [19], we tested three Confirmatory Factor Models (*CFA*) using a maximum likelihood estimation of the parameters. In Model 1, the five types of self-efficacy were considered as separate constructs correlated on the first level. In Model 2, SE_Anger, SE_Sadness and SE_Fear were modelled as expressions of a second-order latent factor reflecting the capability to manage basic negative emotions, whereas SE_Shame and SE_Guilt were modelled as expressions of a different latent second-order factor reflecting the capability to manage self-conscious/moral emotions. In Model 3, SE_Anger and SE_Sadness were grouped as expressions of a second-order latent factor, SE_Fear was left by itself, and SE_Shame and SE_Guilt were grouped together as another second-order factor. In both Model 2 and Model 3, we included a latent third-order factor of a general capability to manage negative affect (SE_Negative Affect) on which the second-order factors loaded. These three models were the most competitive of those tested in a previous study, albeit not in a Spanish context [19].

(b) Some authors have argued for CFI values to be close to .95 rather than .90 (Hu & Bentler, 1999). To me, .90 seems like a rather low bar – maybe the authors want to discuss this.

**Our response:**

**We acknowledge that the reference we used to refer to the CFI threshold was old and thus, we now include the reference of Hu & Bentler (1999) [46]. In addition, we have now included the following sentence in the text:** Although the CFI did not pass the cut-off of .95, it was very close to that threshold, and all the other fit indices were indicative of good fit [35] **(See page. 11).**

(c) It was not clear to me, why the authors did not test three-way interactions (MNESRES x age x sex).

**Our response:**

**We only tested two-way interactions because we originally set out to examine the factorial structure of the MNESRES in terms of different age groups and gender, and to study the construct validity of this scale in relation to various outcomes by taking into account the potential modulation by gender or age. Although we did not examine the suggested three way interactions, we do not doubt that it would be worthwhile to study this should there be access to a larger and representative population.**

(d) It was not clear to me why the authors tested the different linear regression models the way they did. It seemed to me that they wanted to show that although some of the MNESRES facets did not have predictive value above and beyond the other facets, there were some bivariate associations. Two options occur to me: (1) reporting a bivariate correlation table (which I would suggest anyway), and/or (2) reporting regression coefficients for each individual MNESRES facet (controlling for age and sex in each model) & for all combined facets. Also, I would like to see the full models and not just the interaction effects in Step 3. The presentation of results in Table 5 seems slightly misleading to me as the effect of SE_SE of course turns insignificant when adding the predictors in Step 3 (third set of HRA).

**Our response:**

**We agree that the set of hierarchical regressions reported in the original draft may have been confusing. Thus we decided to retain only the first and the most meaningful one: the regression analysis that includes all five MNESRES facets in the same step. In addition, we now present a correlation matrix as suggested by the reviewer (see Table 4 in the revised version of the manuscript, page 18).**

(4) Results

(a) CFA model comparisons: Please note that I am not an expert on SEM. I did not understand why the authors chose Model 2 (correlated factors) instead of a model with a superordinate factor. I see that the AIC was slightly lower in the former model. However, on theoretical grounds, it seems to me that the assumption is that the different factors are part of one underlying construct, namely, self-regulatory efficacy in managing negative emotions. It seems so much more parsimonious when predicting other outcomes to only have one score (i.e., the superordinate score); in particular, when the outcome is as broad as NA (i.e. not at the same level of discrete emotions) or SWL. I would be curious how reliable and predictive an aggregated score would be.

**Our response:**

**We tested several models and decided to report the models that were the most competitive when fitted to the data, as seen previously (Caprara et al. 2013). In the revised manuscript the text now reads (see pages 9 and 10):**

In order to investigate the factorial structure of the MNESRES, in accordance with previous findings [19], we tested three Confirmatory Factor Models (*CFA*) using a maximum likelihood estimation of the parameters. In Model 1, the five types of self-efficacy were considered as separate constructs correlated on the first level. In Model 2, SE_Anger, SE_Sadness and SE_Fear were modelled as expressions of a second-order latent factor reflecting the capability to manage basic negative emotions, whereas SE_Shame and SE_Guilt were modelled as expressions of a different latent second-order factor reflecting the capability to manage self-conscious/moral emotions. In Model 3, SE_Anger and SE_Sadness were grouped as expressions of a second-order latent factor, SE_Fear was left by itself, and SE_Shame and SE_Guilt were grouped together as another second-order factor. In both Model 2 and Model 3, we included a latent third-order factor of a general capability to manage negative affect (SE_Negative Affect) on which the second-order factors loaded. These three models were the most competitive of those tested in a previous study, albeit not in a Spanish context [19].

**In addition, in the comparison of the CFA model, according to the standard procedure to evaluate the model’s fit proposed by Kline (1988), we chose Model 1 (the five-factor oblique model) over Model 2 and 3 (including second order factors), because the indices were slightly better and the AIC was lower in Model 1 than in Model 2 and 3.**

(b) Interaction effects in the prediction of SWL (regression): The interpretation of the interaction effects does not seem quite right to me. The interaction effects show differences between male and female participants. I am not sure how the data was coded, but assuming that men = 0 and women = 1, self-efficacy for regulating fear was more strongly associated with SWL for men than for women, and the other way around for shame. Please check and report the coding in the manuscript.

**Our response:**

**We apologize that the interaction effects were unclear. The data were coded as men=0 and women = 1, as indicated in the legend to Table 5 in the revised version of the paper (see page 20). The Reviewer’s interpretation of the significant interaction effects is correct and as such, we have modified the text to make this clearer (see page 19):**

“We plotted simple slopes of the aforementioned self-efficacy beliefs using high and low values (above and below the median) and accordingly, we found life satisfaction was particularly high in men with high SE_Fear relative to both men and women with low SE_Fear (Figure 2A). In addition, life satisfaction was particularly higher in women with high SE_Shame than in both men and women with low SE_Shame (Figure 2B), as well as in men with high SE_Shame”.

(5) Discussion

(a) The authors state that they found support for the reliability and validity of the scale. However, while the authors investigate the factorial structure, the reliability of the measure was not tested (in terms of internal consistency or re-test reliability). In terms of validity, the authors tested the predictive validity, but not convergent and discriminant validity, for example. Therefore, the authors may want to be more specific with their conclusions.

**Our response:**

**The internal consistency coefficients are presented on page 12 of the manuscript. The data regarding the predictive validity and the construct validity, are offered based on the analysis of the relationships between dimensions and criteria (NA and SWL) using the CFA, and that of the dimensional structure, respectively. The discussion has been revised accordingly.**

(b) I do not understand why the authors conclude that “one may surmise that the common distinction between basic and self-conscious/moral emotions holds good also for self-efficacy beliefs relating to the management of those emotions.” (p. 27). If that was the case, shouldn’t the factor structure be different (i.e., two factors)?

**Our response:**

**We believe that this distinction holds because the model tracing a common SE_Guilt and SE_Shame factor is still valid, and because they are associated with Negative Affect and Life satisfaction differently than SE_Anger, SE_Sadness and SE_Fear.**

(c) I would have wanted the authors to discuss the fact that guilt and shame had no predictive value above and beyond the other facets. One hypothesis that came to my mind was that these emotions may be relatively rare in healthy adults’ everyday life?

**Our response:**

**In revising the discussion we have underlined the importance of maintaining the distinction between basic and self-conscious/moral emotions, and the uniqueness of each discrete emotion. Likewise, we point to the different impact of self-efficacy beliefs related to basic emotions and to self-conscious/moral emotion on negative affect and life satisfaction. We are aware of the limitations of self-reports in general, and do not ignore that self-reports about intimate feelings of guilt and shame are particularly sensitive to social desirability, as well as to various kinds of self-regulatory mechanism that may interfere with their reliability.**

(6) Other comments

(a) Sometimes, sign. results are presented in bold print in the tables, and sometimes not. In Table 4, the estimate for the SE_G X Sex interaction is printed in bold although it is not sign (outcome SWL).

**Our response:**

**We apologize for this confusion, the tables have now been revised and they have been modified accordingly (see Table 5, page 20).**

(b) I was not entirely sure whether mastery beliefs and self-efficacy were used synonymous here.

**Our response:**

**We feel that they are indeed synonymous.**

(c) The data (excel) is not sufficiently labeled. Also, labeling in English would be appreciated.

**Our response:**

**We apologize for this oversight and we now provide a database with the variables labeled in English.**

***Reply to the issues raised by reviewer*** #2:

The researchers addressed three aims: (1) examine measurement invariance of the MNESRES across gender and age in a Spanish sample; (2) examine gender and age differences in self-regulatory self-efficacy beliefs using the MNESRES; and (3) examine associations between discrete emotion self-regulatory self-efficacy beliefs, negative affect, and life satisfaction. These are interesting and important aims and I commend the authors for this study. However, I think several aspects of the presentation and analyses could be improved. I have outlined my specific concerns and suggestions below.

General Writing:

1. The clarity and organization of the manuscript could be improved. In general, the sentences are very long and the paragraphs are rather short. This made it difficult for me to follow the authors' logic from one idea to the next in many locations.

2. There are grammatical errors and inconsistencies throughout the manuscript that should be addressed. For example, individual's is used where individuals' should be used instead in multiple locations. Hyphens in words like life-satisfaction, age-group, and "life-experience" are unnecessary and used inconsistently.

3. In the abstract and in several locations throughout the manuscript, the authors combine all three research aims into a single sentence or phrase. This makes it difficult to understand the key research questions. Instead, the authors should consider stating the three aims separately.

4. The authors should remove the causal language from the Title ("counteract" and "promote").

**Our response:**

**The title has been changed in the revised manuscript, and the abstract has been revised to make it clearer and more concise, removing unjustified causal language. In addition, the text has been proof read by a native English speaker in order to avoid grammatical errors and inconsistencies.**

5. It is unclear throughout whether the basic emotions versus moral emotions distinction is a key research question/guiding motivation of the present research or if it is a secondary exploratory analysis.

**Our response:**

**This is indeed a key question motivating this research.**

6. The manuscript should be carefully proofread and edited for clarity.

**Our response:**

**Please see above**

7. The first sentence of the Introduction is much broader than the topic of the paper. An opening sentence focused on self-efficacy beliefs (rather than theories of personality more generally) would be more appropriate.

8. The Introduction could benefit from clearer organization, perhaps with subheadings or at least specific paragraphs focused on the different components of the research question (e.g., self-efficacy beliefs, the structure of emotion regulation self-efficacy beliefs, the role of discrete emotions, associations between self-efficacy beliefs and well-being outcomes).

**Our response:**

**The introduction has been revised throughout to clarify the theoretical background and the aims of the paper, as recommended by the reviewer. Sub-headings have also been introduced to help make this section clearer.**

9. Several assertions in the Introduction lack references. (e.g., "While people display basic emotions like anger and sadness from the early stages of life, self - conscious/moral emotions do not appear unless self-structures and processes develop..." and "A large body of research has documented that the beliefs people hold about their capacity to cope efficaciously with specific challenges and to face demanding situations, exert a pervasive influence over thought, motivation and action across different domains of functioning.").

**Our response:**

**We have now added the appropriate references**

10. The Introduction focused heavily on scale development and is missing relevant literature to links between emotion regulation self-efficacy beliefs and well-being outcomes. This seems like an important literature to review, given that it is a key focus of the present research. Relatedly, the authors should consider reviewing emotion regulation self-efficacy research beyond the MNESRES (e.g., Emotion Regulation Questionnaire - Self Efficacy; Goldin et al., 2009; beliefs about emotions; Ford & Gross, 2019).

11. The following hypothesis seems much too broad to come from the reviewed evidence: “These findings further corroborate the hypothesis postulating that distinct discrete emotions play a different role with regard to adaptation and, as such, they require a specific consideration regarding their assessment and their treatment.” Is this the authors' hypothesis? If so, it should be significantly narrowed. If it is an existing hypothesis from the literature, a reference should be cited and it should be used to support the researchers' focus on discrete emotions rather than the other way around. In other words, this broad hypothesis can motivate the researchers' more specific research question, however, the researchers' specific research question cannot motivate this broader hypothesis.

**Our response:**

**The importance of the distinction between basic and self-conscious/moral emotions with regards self-efficacy beliefs has been made clearer. Accordingly, we have clarified why we might expect different associations of self-efficacy beliefs related to basic and self-conscious/moral emotions with negative affect and life satisfaction (see pages 2-7).**

**Methods and Results**

12. What does "Within such not previously explored age group," refer to? In the next paragraph, the authors note that the scale was previously validated in an Italian sample of young to older adults.

**Our response:**

**The sentence has been modified as follows (see page 6):**

The study was carried out on a large sample that included males and female of different ages. The size and variation in the sample allowed the evaluation of the extent to which self-efficacy beliefs in terms of the individual’s capacity to manage distinct negative emotions is associated with negative feelings and life satisfaction, across ages and gender.

13. The researchers should list all 10 negative emotional states in the PANAS. This is crucial information for interpreting findings given that the research question is about discrete emotions. Readers shouldn’t have to refer to a separate reference to obtain this information. My key question here is, are basic emotions and self-conscious emotions both represented in the PANAS items? Are the five discrete emotions from the MNESRES represented in the PANAS?

**Our response:**

**Yes they are. To facilitate the readers’ understanding of this issue, we now present the items that were mostly associated with the facets of the MNESRES’s in the corresponding measures section (page 9):**

**“Among the ten items included in this score, there were “afraid”, “worried”, “hostile”, “guilty”, “irritable”, “ashamed”.”**

14. The authors need to justify their analytic approach, in particular, the hierarchical regressions. 14a.Why were age AND gender interactions included together? This makes for a more complex model that could obscure significant interactions.

14b. Why were moral emotions and basic emotions included in different steps. Was this based on theory? Was this based on the observed correlations or the result of the first set of regressions? If so, this should be noted.

**Our response:**

**We have now clarified our analytic approach and the hierarchical regressions performed. Age and gender interactions were included to investigate which contributed most to the variance of the dependent variables. After testing several hierarchical models, we retained the regression analysis that included all five of the MNESRES facets in the same step, reporting their correlations matrix (see Table 4 in the revised version of the paper).**

15. Given the focus on basic versus moral emotions, I am surprised the researchers did not test this distinction in the factor analysis. Was there a reason for not testing a model with a basic emotions factor and a moral emotions factor?

**Our response:**

**In fact we tested several alternative models and we decided to retain those that fitted best with the data while corroborating previous findings. As such, we have now modified the text to read:**

In order to investigate the factorial structure of the MNESRES, in accordance with previous findings [19], we tested three Confirmatory Factor Models (*CFA*) using a maximum likelihood estimation of the parameters. In Model 1, the five types of self-efficacy were considered as separate constructs correlated on the first level. In Model 2, SE_Anger, SE_Sadness and SE_Fear were modelled as expressions of a second-order latent factor reflecting the capability to manage basic negative emotions, whereas SE_Shame and SE_Guilt were modelled as expressions of a different latent second-order factor reflecting the capability to manage self-conscious/moral emotions. In Model 3, SE_Anger and SE_Sadness were grouped as expressions of a second-order latent factor, SE_Fear was left by itself, and SE_Shame and SE_Guilt were grouped together as another second-order factor. In both Model 2 and Model 3, we included a latent third-order factor of a general capability to manage negative affect (SE_Negative Affect) on which the second-order factors loaded. These three models were the most competitive of those tested in a previous study, albeit not in a Spanish context [19].

16. The abbreviations for the different discrete emotions are hard to remember. The authors should consider using more descriptive abbreviations or the complete terms (e.g. SE_fear).

**Our response:**

**We have changed the abbreviations for discrete emotions throughout the paper, as such: anger/irritation is now abbreviated to SE_Anger; sadness to SE_Sadness; fear to SE_Fear; shame to SE_Shame; and guilt to SE_Guilt.**

Discussion:

17. I was surprised that the authors did not discuss the differential associations of discrete emotions versus moral emotions with well-being, given the focus on this in the Introduction and Results. Could these results be explained simply by the fact that basic emotions occur more frequently and/or are better represented in the PANAS?

**Our response:**

**This issue has been largely addressed in the revised discussion:**

In addressing negative emotions, we did not exclude that they could be traced to a hierarchical model. However, the present and previous findings invite us to be cautious about the extent to which the regulation of negative emotions can be traced to a common sense of mastery. Currently, the structure of self-efficacy beliefs assessed by the MNESRES parallels the common distinction between basic emotions like anger, sadness and fear, and that of self-conscious moral emotions like shame and guilt. The results of the CFA indicate they are correlated no less than they are distinctive. Thus, one may surmise that the common distinction between basic and self-conscious/moral emotions also holds well for self-efficacy beliefs in terms of the management of those emotions. It is also likely that the regulation and the beliefs of mastery of basic emotions and of self-conscious/moral emotions rely on mechanisms and strategies that differ from one to the other.

***Reply to the issues raised by reviewer***  #3:

The goal of the present study was to how people’s efficacy beliefs about managing negative emotions impact experienced negative affect and well-being in an adult Spanish sample. In particular, the authors aim to establish measurement invariance in the Spanish sample across age and gender, and examine construct validity of the measure in a Spanish sample by relating the MNESRES to outcomes of adaptation. The study is well-conducted, the analyses rigorous, and the conclusions sound. There are only some minor changes I would recommend, as detailed below:

**INTRODUCTION:**

The first paragraph discusses social cognitive theory of personality, which feels a bit off without first introducing emotional regulatory self-efficacy. A re-ordering of this paragraph would help the reader make the connection between social cognitive theory of personality and the primary focus of the paper—emotional regulatory self-efficacy—more readily. The introduction overall seems to focus very specifically on emotion regulation self-efficacy scale with one group of researchers. A broader discussion of how this measure fits into the literature on emotion regulation and self-efficacy would be helpful. For example, Ford & Gross 2019 Current Directions paper discusses this in some detail (see references).

**Our response:**

**The introduction has now been fully revised and reorganized, including new sub-sections and adding new references.**

METHODS:

Line 174: Please add example items for the MNESRES. Why is chronbach’s alpha not reported? Please add this info in.

**Our response:**

**We present an item as an example for each dimension of the MNESRES in the description of this tool, as well as their Chronbach’s alpha value (see “Confirmatory Factor Analyses and Reliabilities” section: pages 9 and 12).**

Line 224, 229, and 233: Regressions instead of regression.

**Our response:**

**We changed “regression” to “regressions” as indicated.**

Line 264, 286-287, 296-297: Please provide the exact item wording.

**Our response:**

**As suggested we provide the exact wording of the item in the text (see pages 12 and 14).**

DISCUSSION:

Discussion is well-written and thorough. However, more discussion of why negative self-conscious emotions didn’t relate to outcomes would strengthen the discussion. Furthermore, there is a lack of discussion about how positive emotions efficacy beliefs might explain the lack of findings for negative self-conscious emotions. For example, there is some evidence that positive self-conscious emotions, such as pride, may act as a mediator between self-efficacy beliefs and depression (Van Doren et al., 2019). The lack of inclusion of measures of positive self-conscious emotions may have impacted the findings

**Our response:**

**The discussion has been revised in accordance with thee reviewer’s recommendation (see pages 21-25 in blue):**

See also the response to reviewer 1:

**We do not exclude that self-efficacy beliefs about negative emotions are associated to positive affect. This, however, should be the target of another investigation. In this regard one should take advantage of present findings and of previous findings showing how self-efficacy beliefs about the management of positive emotions are associated to positive affect (Gerbino et al 2016), to further examine how self-efficacy beliefs about positive and negative emotions operate in concert.**

References:

Ford, B. Q., & Gross, J. J. (2018). Emotion regulation: Why beliefs matter. Canadian Psychology, 59, 1–14. https://doi.org/10. 1037/cap0000142

Ford, B. Q., & Gross, J. J. (2019). Why beliefs about emotion matter: An emotion-regulation perspective. Current Directions in Psychological Science, 28, 74–81. https://doi.org/10.1177/0963721418806697

Van Doren, N., Tharp, J. A., Johnson, S. L., Staudenmaier, P. J., Anderson, C., & Freeman, M. A. (2019). Perseverance of effort is related to lower depressive symptoms via authentic pride and perceived power. Personality and Individual Differences, 137, 45–49.
