# Supplementary material for: How self-efficacy beliefs in dealing with negative emotions are associated to negative affect and to life satisfaction across gender and age
Source: PLoS One. 2020 Nov 18;15(11):e0242326. doi: 10.1371/journal.pone.0242326 (PMC7673490; doi:10.1371/journal.pone.0242326)
Supplement: Attachment — Submitted filename: Response to Reviewers.docx [file pone.0242326.s007.docx]

Reviewer #1: I read the manuscript for the second time. The revised version is much more concise and much easier to follow. I also very much appreciate that the authors now provided the data with the relevant variables labeled in English. However, I still have a few concerns.

(1) Regulation of basic emotions vs. moral emotions: I still had some difficulty following the argument that the regulation of basic emotions should be more important than the regulation of moral emotions. For parts of the argument, references to relevant research findings seem to be missing. For example, the authors write (pp. 5-6): “Indeed, negative basic emotions like anger, fear and sadness have a pervasive impact over individuals’ life, and their regulation has a primary influence on adaptation when compared to self-conscious/moral emotions like guilt and shame.” I think such a statement (and similar statements in the manuscript) would need a reference.

**Our response:**

**In make our hypothesis clearer, we have added the following considerations to the text (see page 6):**

In terms of the regulation of basic emotions vs. self-conscious/emotions, our reasoning was based on two considerations:

1) Basic emotions are fundamental for the individual’s survival and as such, they are largely prewired in our brain, manifesting themselves early in development and with similar features across cultures. By contrast, moral emotions appear later in development, and they rely upon the appearance of cognitive structures that underlie the formation of a self- system to which they are instrumental and that maintains behavior within the limits of social and personal standards. Moreover, these emotions are largely conditional, and they are shaped by social experiences and cultural references (Lewis, 1993; 2014; Saarni, Campos, Camras & Witherincton, 2006).
2) Basic negative emotions can be viewed as precursors of self-conscious/ emotions on which reactions to harm and pleasure are founded. While negative basic emotions may represent survival responses to external harm or danger in the outside world, self-conscious/ moral emotions underlie self-respect and they respond to violations of internal oughts and beliefs (Caprara & Vecchione, 2017).

**We would just like to clarify that it was not our intention to suggest that the regulation of basic emotions is more important than the regulation of self –conscious/moral emotions but rather, that the former exerts a stronger primary influence on adaptation than the latter.**

**We apologize for not having made this issue clearer, however we wanted to emphasize that during the course of development, the regulation of negative basic emotions condition the emergence and regulation of self-conscious/moral emotions (this reasoning is based on Caprara & Vecchione, 2017). As such, we do not rule out the possibility that the regulation of moral/self conscious emotion may even exert a stronger influence on an individual’s well-being than the regulation of basic emotions, as this remains to be proven. Instead, we believe that the capacity to regulate basic emotions precedes and conditions the development of the capacity to regulate self conscious/moral emotions.**

- Saarni, C., Campos, J. J., Camras, L. A., & Witherington, D. (2006). Emotional development: Action, communication, and understanding. In W. Damon, R. M. Lerner, & N. Eisenberg (Eds.), Handbook of child psychology: Vol. 3. Social, emotional, and personality development (6th ed., pp. 226–299). New York: John Wiley

Lewis, M. (1993). “Self-Conscious Emotions: Embarrassment, Pride, Shame and Guilt.” In M. Lewis, & J. M. Haviland (Editors) Handbook of Emotions (pp. 563-594). New York: Guilford.

Lewis, M. (2014). The rise of consciousness and the development of emotional life. New York: Guilford Press.

(2) Reliabilities of the MNESRES subscales were low for anger and sadness (.55, .54). This could be discussed.

**Our response:**

**We have now addressed this issue in the discussion (See page 25):**

**“The alpha values of the MNERES subscales for anger and sadness are far from optimal indices and this represents an important limitation. We are inclined to believe that this is due to the limited number of items, although this issue should be addressed in future studies by either enlarging the number of items or through their reformulation.”**

(3) Figure 1: Some correlations between factors seem to be missing from the figure (e.g., correlations between SE_G and SE_DS.

**Our response:**

**We thank the reviewer very much for bringing this error to our attention. We have now uploaded an updated version of Figure 1 in which we have added the two correlations that were missing from the original version, namely the correlation between SE_AI and SE_SE (r = 0.47) and between SE_DE and SE_G (r = 0.50).**

(4) Interaction effects/ Figure 2: I still think that the authors’ interpretation of the interaction effects is (a) not really in line with their research question and (b) not 100% correct. With regard to (a), I think what the authors did (and illustrated), is considering the SEs as moderators of the association between sex and satisfaction with life. However, since the research is concerned with associations between the SEs and satisfaction with life, it makes much more sense to me to consider sex as the moderator here. Statistically, there is of course no difference between considering sex or the SEs as moderators. However, the interpretation is slightly different. The results show that there is an association between SE_fear and life satisfaction: Individuals with higher SE_fear have higher life satisfaction. Treating sex as a moderator, the interaction effect (SE_fear x sex, b= -.12) means that for women, the association (semi-partial correlation) between SE_fear and life satisfaction is weaker than for men. Although there is no main effect of SE_shame on life satisfaction, there is an interaction effect SE_shame x sex (b=.14), indicating that for women, SE_shame is related to satisfaction with life, but not for men.

With regard to (b), the authors write: “life satisfaction was particularly higher in women with high SE_Shame than in both men and women with low SE_Shame (Figure 2B), as well as in men with high SE_Shame.” This cannot be concluded from the interaction effect, which only indicates that there are differences in trajectories. One would have to test these specific differences between higher and lower groups post-hoc. In the plots presented in the paper, it is fairly obvious that women high in SE_shame are not more satisfied with their life than men with high SE_shame (the difference is only about 0.1 points on the scale). As sex is a categorical variable here, I also do not think that plotting the data as trajectories in the way presented in the paper is most useful. Therefore, I would strongly suggest to switch to considering sex as the moderator, and changing the interpretation and the plots accordingly.

**Our response:**

**We thank the Reviewer for his/her efforts and guidance to help us strengthen the interpretation and the discussion of the results of our study, favoring a straightforward future interpretation of our results by its readers. In line with Reviewer’s comments, we are also confident that our decision to examine the moderating role of sex in the association between self-efficacy beliefs in dealing with negative emotions and life satisfaction will be of use in the future. We appreciate the efforts of the reviewer to consider our results from an alternative perspective, and we are grateful for the suggestion to assess the correlation between SE_F and life satisfaction, and between SE_SE and life satisfaction, separately for men and women. As a result, we found that the association between SE_F and life satisfaction for men is r = 0.36 and for women was r = 0.23 (p<.001). In addition, the association between SE_SE and life satisfaction for men was r = 0.14 and r = 0.23 for women (p<.001).**

**Those results are fairly consistent with the Reviewer’s suggestion and indeed, “for women, the association (semi-partial correlation) between SE_fear and life satisfaction is weaker than for men”. Whereas, we did not find that “for women, SE_shame is related to satisfaction with life, but not for men”, it did appear that for women the association between SE_fear and life satisfaction is stronger than for men.**

**As a result, we have addressed this issue in the main text by adding the following sentence to page 19:**

“we found that the association between life satisfaction and SE_Fear was stronger for men than for women (Figure 2A). In addition, the association between life satisfaction and SE_Shame was stronger for women than for men (Figure 2B).”

**Regarding the second part of this comment, as we did not test these specific differences between higher and lower groups post-hoc, we agree that it was not correct to suggest that “life satisfaction was particularly higher in women with high SE_Shame than in both men and women with low SE_Shame (Figure 2B), as well as in men with high SE_Shame”). Thus, we decided to remove that sentence from the manuscript. In addition, and as suggested by the Reviewer, we have adjusted our plots to more clearly support our interpretation that sex is a moderating variable in our analyses.**

**(5) Discussion: I** think that some statements made by the authors in the discussion need further elaboration. I also felt that some conclusions go beyond what was studied.

(a) “Albeit with caution, it may be concluded that the five-oblique-factor solution further corroborates the view that self-efficacy beliefs are associated with the regulation of discrete emotions**.” (p.20)** Since the questionnaire measures self-efficacy beliefs with regard to regulating discrete emotions, I am not sure how a factor analysis can corroborate the association between self-efficacy beliefs and regulation. Maybe the authors could rephrase?

**Our response:**

**We have now rewritten this part of the discussion as follows (see pages 21-22):**

**“Albeit with some caution, it might be concluded that the five-oblique-factor solution is in accordance with the underlying specificity of each emotion, implying that it would be recommendable to focus on each emotion irrespective of its source, manifestations and means of regulation. This recommendation may also be worthy of consideration in terms of self-efficacy beliefs, as corroborated by the five factor solution of the confirmatory factor analysis. However, this does not fully exclude attempting to trace one common factor to all five emotional self-efficacy beliefs, since the statistical divide between the two solutions is minimal.”**

(b) “**However, the present and previous findings invite us to be cautious about the extent to which the regulation of negative emotions can be traced to a common sense of mastery.”** (p. 21) – In the present study, a model with one common mastery factor was not tested. The fact that a model with five correlated factors fit the data best does very much invite the idea that there may be a common factor, I think. I still think that the best option here would be to simply test a model with a common factor. As a reader of this paper, I would be very interested in that.

(c) “**Thus, one may surmise that the common distinction between basic and self-conscious/moral emotions also holds well for self-efficacy beliefs in terms of the management of those emotions.” (p.21).** The reply given by the authors regarding my question in the first review does not convince me. I still fail to see how the present study supports this conclusion. Based on the CFAs, a model with 5 correlated factors fit the data best, which is a model that does not support this distinction. I understand that the fit of alternative models was also not that bad, but I do not think that this is strong evidence to corroborate a distinction. In fact, the correlations between the subscales (Table 4) show that the correlations between SE_shame with SE_sadness and with SE_fear were similar to the correlation between SE_anger and SE_fear, which does not indicate that SE_shame belongs to a different factor than the other SEs. Furthermore, all factors correlated with the outcomes (negative affect and satisfaction with life). The fact that SE_shame and SE_guilt were not uniquely predictive of the outcomes above and beyond all other factors is not indicative of these factors not belonging to the same underlying construct. It may very well be the case that the distinction makes sense conceptually and that this was found in other studies, but in the present study, the data does not support such a conclusion, I would say.

**Our response:**

**Regarding the issues raised in b and c:**

**We have now added the results for the Model with one second order factor overarching the five first order factors, as proposed by the Reviewer (please see the revised text on pages 10 and 12). Indeed, the model with five oblique factors is that which best fits the data.**

**We do not underestimate the points made by the reviewer and we agree that future studies may lead us to different conclusions. However, previous and present findings are consistent with our theoretical reasoning (see above), sustaining the distinction between basic and self-conscious/moral emotions. We believe that this distinction is not only relevant conceptually but also, heuristically and practically. On the one hand it encourages us to investigate how the management of basic emotions may depend on and condition the management of self-conscious/moral emotions. On the other hand, it suggests that equal attention should be paid to what is common and specific to emotional experience, and thus, to design interventions accordingly. The progress made by Bandura and Caprara in terms of tasks specific to domain specific mastery beliefs has lead us to first distinguish between mastery beliefs regarding positive and negative emotions, and then, to focus on negative emotions that try to balance specificity and generality (Bandura et al. 2003, Caprara, 2002).**

(d) “One might think that most people learn how to refrain from anger earlier” (p.21-22) – Why might one think that? (see Comment #1)

**Our response:**

**To clarify this issue, we have added the following text to the Discussion (see page 24):**

**“Children probably learn how to refrain from anger earlier through socialization practices that sustain empathic concern and that lead them to avoid reactions that may hurt others. By contrast, sadness and fear are insidious experiences ……**

Reviewer #2:  The authors addressed the majority of comments raised in the prior round of revision. I appreciated that the authors tempered their conclusions about the factor structure and direction of causality in the Discussion section. I also appreciated that they considered both evolutionary and constructivist views of emotion in the Introduction. Overall, this is a thorough revision of a nice manuscript. I have outlined a small number of minor suggestions below.

1. The rationale for the hypothesis that regulation of basic emotions should be more important for well-being (bottom of page 5, top of page 6) was not clear.

**Our response:**

**In make our hypothesis clearer, we have added the following considerations to the text (see page 6):**

In terms of the regulation of basic emotions vs. self-conscious/moral emotions, our reasoning was based on two considerations:

1) Basic emotions are fundamental for the individual’s survival and as such, they are largely prewired in our brain, manifesting themselves early in development and with similar features across cultures. By contrast, moral emotions appear later in development, and they rely upon the appearance of cognitive structures that underlie the formation of a self- system to which they are instrumental and that maintains behavior within the limits of social and personal standards. Moreover, these emotions are largely conditional, and they are shaped by social experiences and cultural references (Lewis, 1993; 2014; Saarni, Campos, Camras &Witherincton, 2006).
2) Basic negative emotions can be viewed as precursors of self-conscious/moral emotions on which reactions to harm and pleasure are founded. While negative basic emotions may represent survival responses to external harm or danger in the outside world, self-conscious/ moral emotions underlie self-respect and they respond to violations of internal oughts and beliefs (Caprara & Vecchione, 2017).

**We would just like to clarify that it was not our intention to suggest that the regulation of basic emotions is more important than the regulation of self –conscious/moral emotions but rather, that the former exerts a stronger primary influence on adaptation than the latter.**

**We apologize for not having made this issue clearer, however we wanted to emphasize that during the course of development, the regulation of negative basic emotions condition the emergence and regulation of self-conscious/moral emotions (this reasoning is based on Caprara & Vecchione, 2017). As such, we do not rule out the possibility that the regulation of self-conscious/moral emotion may even exert a stronger influence on an individual’s well-being than the regulation of basic emotions, as this remains to be proven. Instead, we believe that the capacity to regulate basic emotions precedes and conditions the development of the capacity to regulate self-conscious/moral emotions.**

- Saarni, C., Campos, J. J., Camras, L. A., & Witherington, D. (2006). Emotional development: Action, communication, and understanding. In W. Damon, R. M. Lerner, & N. Eisenberg (Eds.), Handbook of child psychology: Vol. 3. Social, emotional, and personality development (6th ed., pp. 226–299). New York: John Wiley

Lewis, M. (1993). “Self-Conscious Emotions: Embarrassment, Pride, Shame and Guilt.” In M. Lewis, & J. M. Haviland (Editors) Handbook of Emotions (pp. 563-594). New York: Guilford.

Lewis, M. (2014). The rise of consciousness and the development of emotional life. New York: Guilford Press.

2. Please name all 10 emotions that were included in the negative emotion measure. This information is important for evaluating the degree of overlap between the self-efficacy beliefs measure and the negative emotion measure.

**Our response:**

**As suggested by the reviewer, we have now named all 10 emotions that were included in the negative emotion measures (see page 9):**

**“For the purposes of this research, we used the Spanish version of this scale [43], and the emotions referred to as “distressed”, “upset”, afraid”, “scared”, “nervous”, “jittery”, “hostile”, “guilty”, “irritable” and “ashamed” were** **the ten items included in this score”.**

3. It is unclear what is meant by this sentence in the Discussion: "*Since the sample was taken mostly from a relatively affluent middle class population, one cannot exclude significant variations due to age and gender, either in the means or in the relationship of the variables examined, particularly among a population whose conditions of life are quite different*." I believe the authors are trying to make a point about lack of generalizability beyond middle class population, but it is unclear to me what they are saying about age and gender. **Pag 23**

**Our response:**

**We agree that this phrase is confusing and does not add anything, and as such we have now removed it from the manuscript (page 25).**

4. The first paragraph includes jargon and complex sentence structures. This may be personal preference and the authors may feel free to leave this paragraph as is, however I found it difficult to understand.

**Our response:**

**We have reconsidered our introductory sentence as recommended by the reviewer and despite the reviewer’s reservations, we would prefer to leave this sentence as it is. We believe that this sentence conveys what we believe to be distinctive of the social cognitive approach to personality: namely a system made by psychological structures that emerged over the course of development to operate together in order to produce balanced growth and adaptation.**
